# Medicine Storage and Dispensing Facilities in Public Healthcare Pharmacies of Puducherry, India

**DOI:** 10.7759/cureus.21389

**Published:** 2022-01-18

**Authors:** Dinesh K Meena, Mathaiyan Jayanthi, Kesavan Ramasamy, Mahalakshmy Thulasingam

**Affiliations:** 1 Pharmacology, Jawaharlal Institute of Postgraduate Medical Education and Research, Puducherry, IND; 2 Preventive & Social Medicine, Jawaharlal Institute of Postgraduate Medical Education and Research, Puducherry, IND

**Keywords:** good drug-dispensing practices, who, primary health care, pharmacist, storage

## Abstract

Background

Large amounts of medicines are wasted during procurement, storage, distribution, and utilization. Proper procurement, storage, dispensing, and documentation of medicines are important aspects of pharmacy management. The World Health Organization (WHO) and the Indian Pharmaceutical Association (IPA) have developed guidelines for the storage and dispensing of medicines by pharmacists. This study was conducted to assess the storage and dispensing facilities of medicines in public healthcare pharmacies of Puducherry province in south India.

Methodology

A one-time survey was conducted in 10 public healthcare pharmacies by filling the checklist which was prepared based on the WHO and IPA guidelines.

Results

Facilities such as adequate surface area, storage area, reception area, and availability of water supply in dispensing area were available in 90% of surveyed pharmacies. The most common system used for the arrangement of medicines was alphabetical order (70%). In 80% of pharmacies, a sufficient number of shelves was available for storage of medicine, and in 90% of pharmacies, shelves were properly labeled. None of the pharmacies had separate storage facilities for expired medicines and narcotic drugs.

Conclusions

In Puducherry, pharmacy services are provided by qualified and experienced pharmacists. Although most of the surveyed pharmacies had all the required infrastructure and equipment facilities, few pharmacies need to improve their facilities to promote good drug-dispensing practices.

## Introduction

Appropriate use of medicines in the pharmacy department is a multidisciplinary responsibility under the supervision of a qualified pharmacist and includes procurement, storage, preparation, and dispensing [1]. The main responsibilities of a pharmacist include compounding and dispensing medicines and providing counseling to the patients. Proper procurement, storage, dispensing, and documentation of medicines are important aspects of pharmacy management [2]. Large amounts of medicines are wasted during procurement, storage, distribution, and utilization. The World Health Assembly organized two meetings in Delhi in 1988 and Tokyo in 1993 on the role of pharmacists in the safe use of medicines (WHO/PHARM/94.569) [3]. In 1992, the International Pharmaceutical Federation (FIP) developed standards for pharmacy services, namely “Good Pharmacy Practice in Community and Hospital Pharmacy Settings,” based on the pharmaceutical care provided by pharmacists. The guidelines set national standards for pharmacists’ activity for promoting health, patient self-care, supply of medicines and medical devices, and improving prescribing and medicine use [4]. A pharmacy in India should maintain and follow standard operating procedures [5]. A good dispensing environment is an important part of day-to-day pharmacy practice and includes staff, physical surroundings, and equipment. Most medical products are for internal use and require a clean, hygienic, and organized dispensing environment to prevent contamination. Proper environmental controls such as temperature, light, humidity, conditions of sanitation, ventilation, and segregation must be maintained, along with warehouses offering sufficient storage space. Warehouses should have sufficient storage space along with the necessary facilities to handle medicines efficiently and as per the guidelines. Storage must be secure, and fixtures and equipment used to store medicines should be constructed in such a way that medicines are accessible only to designated and authorized personnel. Such personnel must be carefully selected. Safety is an important factor, and proper considerations should be given to the safe storage of poisons (disinfectants or other chemicals not meant for human use) and inflammable compounds. A pharmacy should have all the necessary equipment required for good drug-dispensing practices [5,6].

Public health facilities remain the main source of healthcare services for the majority of individuals. The rational use of medicines in public health facilities is directly dependent on pharmacy services. Therefore, we conducted this study to assess medicine storage and dispensing facilities in public healthcare centers of a south Indian Union Territory.

## Materials and methods

Study design

A one-time survey was conducted to assess the facilities for storage and dispensing of medicines in 10 public health care pharmacies of Puducherry.

Study site and duration

This study was conducted between March 2019 and February 2020 in Puducherry. Pondicherry is a union territory of India and consists of four districts, namely, Puducherry, Karaikal, Yanam, and Mahe. This study was conducted in the Puducherry district which is the largest one in Puducherry. Puducherry district has an adequate number of public health facilities functioning under both the central (one tertiary care hospital and two primary health centers [PHCs]) and state government (27 primary health centers, two community health centers, and five district hospitals).

Study population

A sample of 10 public health facilities was selected based on their geographical location. The sample consisted of nine PHCs and the medicine outpatient unit of one tertiary care hospital. These nine PHCs were selected randomly based on their geographical location (at least one PHC from north, south, west, and east) and the number of PHCs present in each direction (at least one from an area with 1-5 PHCs, two from an area with 5-10 PHCs, and three from an area with more than 10 PHCs). Thus, we selected nine PHCs (one from north, three from south, three from west, and two from east). Out of the nine PHCs, seven are functioning under state government and two are functioning under the central government.

Facilities surveyed

A checklist of basic infrastructure facilities for proper storage, maintenance, and dispensing of medicine was prepared as per the guidelines of the Indian Pharmacist Association (IPA) [5] and the World Health Organization (WHO) [6]. The checklist consisted of the sociodemographic characteristics of pharmacy professionals, the system used for the arrangement of medicines, basic infrastructure facilities of pharmacy, and basic equipment for storage of medicines.

Data collection

A one-time survey was conducted in each selected health center. Data collection was done by qualified personnel (Doctor of Pharmacy degree). Proper training was given for data collection before the field visit. Before visiting a pharmacy, the checklist was kept confidential to avoid bias. The checklist was filled by observing the pharmacy of individual health facilities in the presence of the pharmacists and medical officers on the day of the survey.

Data analysis

Data were entered in Microsoft excel. Data were separately analyzed for state government and central government pharmacies. The sociodemographic characteristics of pharmacists were expressed as average or percentage. Availability of individual facility/equipment in all surveyed pharmacies was also determined. Data were analyzed using Microsoft Excel.

Ethical approval

The ethical approval for this study was obtained from the Institutional Ethical Committee of Jawaharlal Institute of Postgraduate Medical Education and Research, Puducherry (JIP/IEC/2018/338), India. Administrative approval for collecting data from state government public health facilities was also obtained from the Directorate of Health and Family Welfare, Government of Puducherry (585/DHFWS/PA/2019).

## Results

In this study, we surveyed pharmacy stores of 10 public health facilities to assess basic infrastructure, storage facilities, and equipment required in a pharmacy store. The sociodemographic characteristics of pharmacists showed that all the health facilities had pharmacists, and the average number of pharmacists was 3.2. There were more male pharmacists (53.1%) compared to female pharmacists. Most pharmacists had a Bachelor in Pharmacy (B. Pharmacy) (62.5%), followed by a Diploma in Pharmacy (D. Pharmacy) (25%), only 12.5% of pharmacists had a Master (M. Pharmacy), and none of the pharmacists had a Doctorate (Pharm. D) in pharmacy. All pharmacists (100%) were registered in the state pharmacy council. The average experience of pharmacists was 12.1 years. Detailed sociodemographic characteristics of pharmacists of three types of health facilities surveyed are presented in Table 1.

**Table 1:** Sociodemographic characteristics of pharmacy professionals in surveyed public healthcare pharmacies, Puducherry. n = number of pharmacies surveyed. PHC: primary health center

Sociodemographic characteristics		State govt. PHCs (n = 7)	Central govt. PHCs (n = 2)	Tertiary care teaching hospital (n = 1)	Overall (n = 10)
Average number of pharmacists		1.7	2	16	3.2
Gender (Percentage)	Male	41.6	50	62.5	53.2
	Female	58.4	50	37.5	46.8
Qualification (Percentage)	Pharm. D	0	0	0	0
	M. Pharm	8.3	0	18.5	12.5
	B. Pharm	25	100	81.5	62.5
	D. Pharm	66.7	0	0	25
Registered in state pharmacy council (Percentage )		100	100	100	100
Average experience (in years)		15	10	10.6	12.1

**Figure 1:**
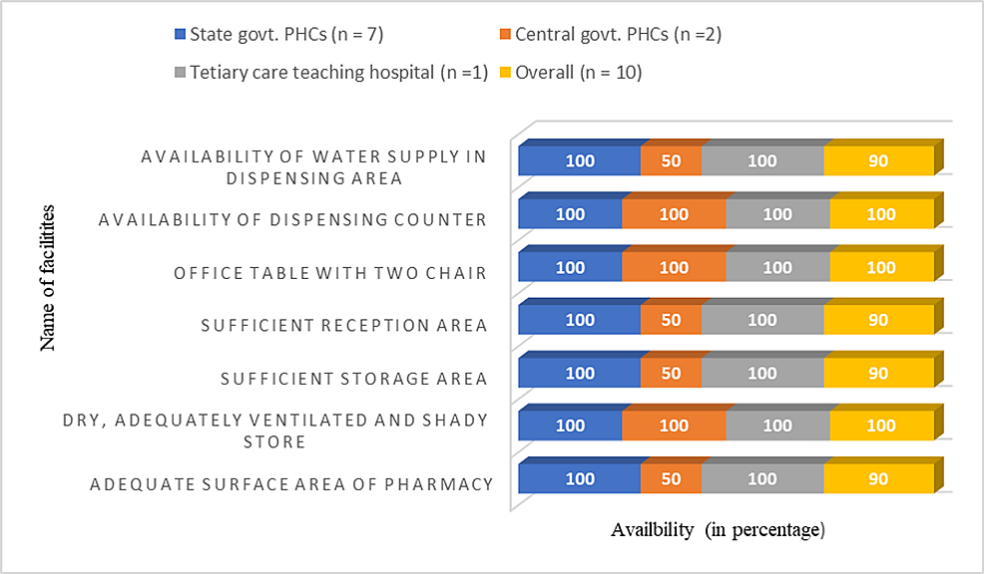
Percentage availability of different infrastructure and storage facilities in surveyed pharmacies. n = number of pharmacies surveyed. PHC: primary health center

We also checked the infrastructure and facilities required for the proper storage of medicines in pharmacies. Facilities such as adequate surface area of pharmacy, sufficient storage area, sufficient reception area, and availability of water supply in dispensing area to wash hands were available in 90% of surveyed pharmacies. Moreover, facilities such as dry and adequately ventilated and shady store, office table with two chairs, and dispensing counter/table were available in all surveyed pharmacies. A complete description of infrastructure and facilities required for the proper storage of medicine in three different types of health facilities is presented in Figure 1.

In this study, we found that the most common system used for the arrangement of medicines was alphabetical order (70%) followed by pharmaceutical-therapeutic order (30%). In 80% of pharmacies, a sufficient number of shelves were available for storage of medicine, and in 90% of pharmacies, shelves were properly labeled. Adequate space for the movement of goods was available in 80% of pharmacies (Table 2).

**Table 2:** Availability of facilities for arrangements of medicines in surveyed public healthcare pharmacies. n = number of pharmacies surveyed PHC: primary health center

Specifications available ( in percentage)		State govt. PHCs (n = 7)	Central got. PHCs (n = 2)	Tertiary care teaching hospital (n = 1)	Overall (n = 10)	
						System followed for arrangement of medicines	Therapeutical order	28.6	50	0	30	
Alphabetical order	71.4	50	100	70		
Pharmaceutical order	0	0	0	0		
No system followed	0	0	0	0		
Sufficient shelves available for storage of medicine		100	50	0	80	
Properly labeled shelves		100	50	100	90	
Adequate space for movement of goods		100	50	0	80	

**Figure 2:**
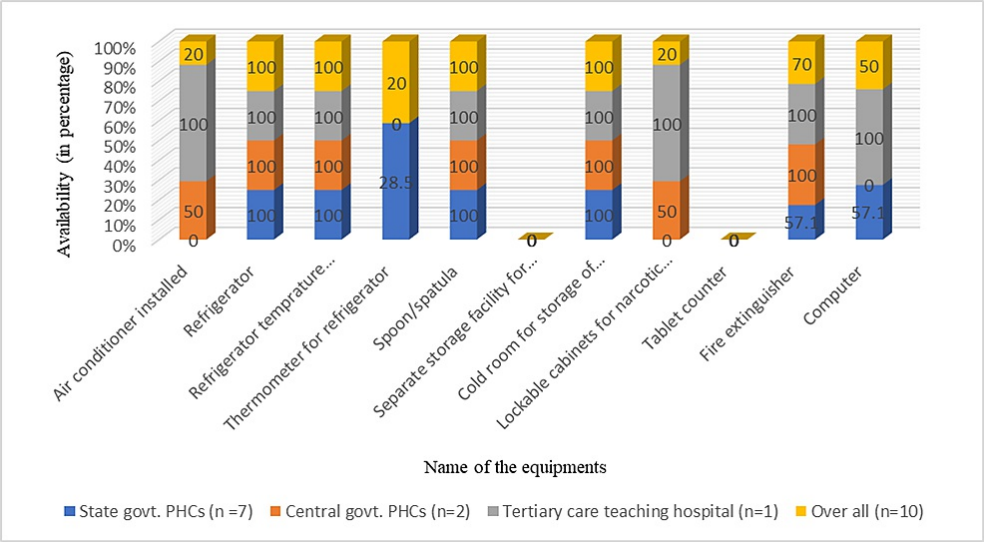
Percentage availability of various equipment for storage of medicines in surveyed pharmacies of Puducherry. n = number of pharmacies surveyed PHC: primary health center

Regarding the assessment of minimum equipment required in pharmacy stores, we found that only 20% of surveyed pharmacies had an air conditioner, thermometer for refrigerator, and lockable cabinets for narcotics and psychotropic substances, while none of the pharmacies had separate storage facilities for expired medicines and tablet counter machines. Overall, 70% of pharmacies had fire extinguishers for safety measures, and only 50% had a computer for the storage of data. Equipment such as refrigerator, spatula/spoon, and cold room to keep vaccines, sera, and biological products were available in all surveyed pharmacies (Figure 2).

## Discussion

In developing countries like India, the majority of the population relies on public health care settings; therefore, it is important to implement good drug-dispensing practices in public health care pharmacies to promote the safe and rational use of medicines. Personnel involved in dispensing medicine and facilities available to store and dispense medicines are important factors that influence dispensing pharmacy practices. Lack of dispensing facilities and dispensing of medicines by an untrained person may lead to wastage and irrational dispensing of medicines. In this study, we found that in all surveyed pharmacies, medicine was dispensed by experienced and registered pharmacists who were following good drug-dispensing practices as well as the pharmacy practice regulations of India. We found that each surveyed health facility had at least one pharmacist, with higher numbers in urban PHCs and tertiary care teaching hospitals. In PHCs, most of the pharmacists had a D. Pharmacy followed by a B. Pharmacy degree and only a few pharmacists had an M. Pharmacy, and none of the pharmacists had a clinical pharmacist (Pharm. D). In a health care setting, pharmacists are mainly responsible for patient counseling regarding the safe use of medicines. In many countries, Pharm. D is the minimum qualification to work as a pharmacist, whereas in India a two-year diploma (D. Pharmacy) is enough to register as a pharmacist, which could be the reason for a high number of D. Pharmacy-qualified pharmacists in PHCs of Puducherry.

Having sufficient surface area of pharmacy is essential for free movement during handling and shifting of medicines. In our study, we found that 90% of pharmacies had an adequate surface area. Pharmacy stores should be dry and adequately ventilated to prevent medicine incompatibility (physical, chemical, and biological) reactions due to dust, moisture, and excessive sunlight. All surveyed pharmacies in this study had a dry, adequately ventilated, and shady store. A pharmacy should have a sufficient storage area to store the bulk quantity of medicines which should be separate from dispensing area. The storage area should be equipped with storage cabinets or racks. Keeping medicines on the floor can lead to contaminations and breakage of medicines. In our study, we found that 90% of pharmacies had sufficient storage area, and 80% of pharmacies were equipped with a sufficient number of shelves for storage of medicine. A pharmacy needs to have a reception area and dispensing counter where a pharmacist can dispense medicines and counsel the patients. A sufficient reception area enables a pharmacist to select, arrange, and label the medicine comfortably. Not having a sufficient reception area can lead to mixing of medicines, improper labeling, and irrational dispensing of medicines. Similarly, a dispensing counter enables patients to communicate with the pharmacist comfortably regarding their medicines. In our study, we found that most of the pharmacies had sufficient reception areas to dispense medicines, and all pharmacies had dispensing counters to counsel patients. Pharmacies should have a water supply to wash hands to prevent contamination by food, different medicines, and in case of breakage of liquid formulations. In our study, we found that nine out of the ten surveyed pharmacies had adequate water supply facilities.

Pharmacists should follow a system (pharmaceutical-therapeutical order, alphabetical order, or pharmaceutical order) of arranging medicines on shelves. Labeling (writing the name of medicine stored) of shelves is also important. Systematic arranging of medicines and labeling of shelves enables pharmacists and other healthcare professionals (in absence of pharmacists) to identify the medicines easily, which not only saves time during dispensing but also prevents the risk of dispensing wrong medicines (especially in the case of medicines sounds alike). In our study, we found that all pharmacists followed orders (70% alphabetical and 30% pharmaceutical-therapeutical order) to arrange medicines on shelves, and in most of the pharmacies (90%), shelves were properly labeled.

Previous studies have shown that storing medicines at a temperature above 25°C can lead to significant reductions in their activity [7]. Exposure of medicines to high temperatures in storage or transport can reduce their efficacy [8]. Therefore, it is important to have an air conditioner in the pharmacy to maintain ideal temperatures, especially in the summer season. Improper storage of medicines at unfavorable temperatures can be one of the important factors that contribute to decreased efficacy of medicines [9]. Therefore, pharmacies should be equipped with facilities such as an air conditioner, a cold room for vaccine storage, and a refrigerator to store medicine and vaccines at the required temperature. We examined the availability of equipment and devices required to maintain an ideal temperature in pharmacy and found that all surveyed pharmacies had cold room facilities to store vaccines, sera, and other biological products, as well as refrigerators to store medicines, while only two out of ten (20%) pharmacies had an air conditioner. Air conditioners were not available in rural healthcare center pharmacies, which could be either because of a lack of budget for the state government health facilities or the cost of purchasing air conditioners might not be considered compared to urban areas. It is important to have a thermometer to monitor the temperature of the refrigerator at a regular time interval. In our study, we found that only two pharmacies had thermometers. As per the Indian Narcotic Drugs and Psychotropic Substances Act, narcotic and psychotropic substances should be stored in lockable cabinets and should be dispensed only by a qualified person. Therefore, we also checked the availability of lockable cabinets for the storage of such substances. We found that only two pharmacies had lockable cabinets. Narcotic and psychotropic drugs are mostly given in tertiary care centers under the supervision of a qualified psychiatric physician and rarely procured at the primary care level which could be the reason for the lack of availability of lockable cabinets at the primary care level. The major limitation of this study is that we only included 10 health facilities of Puducherry.

## Conclusions

In Puducherry, pharmacy services are provided by qualified, registered, and experienced pharmacists who are following regulations prescribed by the WHO/IPA guidelines. Although most surveyed pharmacies had all the required infrastructure and equipment facilities, a few pharmacies lack the facilities such as an adequate storage area, water supply in the dispensing area, sufficient reception area, and lack of separate storage facility for expired medicines, which need to be improved. An educational intervention to update pharmacists on good drug-dispensing practices can improve the storage and dispensing of medicines in Puducherry pharmacies.
